# Laparoscopic Resection of Residual Retroperitoneal Tumor Mass in Advanced Nonseminomatous Testicular Germ Cell Tumors; a Feasible and Safe Oncological Procedure

**DOI:** 10.1038/s41598-019-52109-5

**Published:** 2019-11-01

**Authors:** Çiğdem Öztürk, Lukas B. Been, Robert J. van Ginkel, Jourik A. Gietema, Harald J. Hoekstra

**Affiliations:** 1 Department of Surgical Oncology, University Medical Center Groningen, University of Groningen, Groningen, The Netherlands; 2 Medical Oncology, University Medical Center Groningen, University of Groningen, Groningen, The Netherlands

**Keywords:** Outcomes research, Surgical oncology, Germ cell tumours

## Abstract

Ten-year oncological experience of the University Medical Center Groningen with conventional laparotomy (C-RRRTM) and laparoscopy (L-RRRTM) is described concerning resection of residual retroperitoneal tumor masses (RRTM) in a large series of patients with advanced nonseminomatous testicular germ cell tumors (NSTGCT). 150 consecutive patients with disseminated NSTGCT required adjunctive surgery after combination chemotherapy. L-RRRTM was scheduled in 89 and C-RRRTM in 61 patients. Median residual tumor diameter was 20 mm in the L-RRRTM versus 42 mm in the C-RRRTM group (p < 0.001). Conversion rate was 15% in the L-RRRTM group. Perioperative complications occurred in 5 patients (6%) in the L-RRRTM and 7 (12%, NS) in the C-RRRTM group. Median duration of L-RRRTM was 156 minutes vs. 221 minutes for C-RRRTM (p < 0.001). 17/89 patients in the L-RRRTM group had postoperative complications versus 18/61 patients in the C-RRRTM group (NS). Median postoperative stay in the L-RRRTM group was 2 vs. 6 days in the C-RRRTM group (p < 0.001). During a median follow-up of 79 months, 27 patients had recurrences: 8 (9%) in the L-RRRTM group and 19 (31%) in the C-RRRTM group (p < 0.001). Laparoscopic resection of RRTM for advanced NSTGCT is feasible and an oncologically safe option in appropriately selected patients.

## Introduction

The introduction of platinum-based chemotherapy to treat advanced nonseminomatous germ cell tumor (NSTGCT) has impacted survival rates greatly, with an overall 10-year survival rate of up to 90%^1,2^. Surgery plays a pivotal role in the treatment of residual retroperitoneal tumor masses (RRTM) as well as pulmonary residual disease in NSTGCT and is aimed at resecting viable germ cell cancer tissue and/or teratoma^3–7^. The extent of surgery has remained controversial for many years, with a surgical spectrum varying from a full bilateral retroperitoneal lymph node dissection (RPLND) to a more limited approach with resection of visible abnormal retroperitoneal tumor masses^8,9^. Today’s literature supports that a modified post chemotherapy RPLND, e.g. resection of well-defined residual retroperitoneal tumor masses (RRRTM), is a safe oncological procedure, with less morbidity and it conserves sexual functioning in the majority of these patients^10–12^.

Classically, RPLND or RRRTM was executed through a midline laparotomy. Laparoscopic RPLND (L-RPLND) was first performed in 1992. In the past decade, it has emerged as an alternative to reduce morbidity associated with conventional open surgery using the same boundaries of dissection. Laparoscopic surgery is mainly described in literature for stage I disease as a diagnostic procedure and for the resection of low volume disease^13–16^. In an earlier pilot study, the Groningen study group showed that the laparoscopic approach was feasible, with a low rate of retroperitoneal relapse in advanced testicular cancer in properly selected patients^17^. So far, there are no large consecutive series of disseminated testicular cancer patients described with respect to the results of adjunctive surgery; e.g., conventional versus laparoscopic resection of RRTM. For the laparoscopic resection of RRTM to be considered a safe alternative oncologic procedure compared to conventional open surgery in patients with advanced NSTGCT, long-term follow up assessments in a larger cohort are required.

The current study aimed to describe the 10-year experience of the Comprehensive Cancer Center of the University Medical Center Groningen UMCG with conventional resection of RRTM (C-RRRTM) and laparoscopic resection of RRTM (L-RRRTM) in a consecutive series of patients and compared first the intra and postoperative morbidity data of L-RRRTM group with the C-RRRTM group and secondly focused on long-term oncologic outcome of both surgical procedures. Furthermore, were the oncological and technical boundaries for laparoscopic management in the field of adjunctive surgery after cisplatin combination chemotherapy in patients with testicular cancer defined.

## Methods

The institutional review board of the UMCG was consulted, and they confirmed that no formal written waiver for the need of ethics approval was required because of the retrospective design of the study and anonymised data.

**Figure 1:**
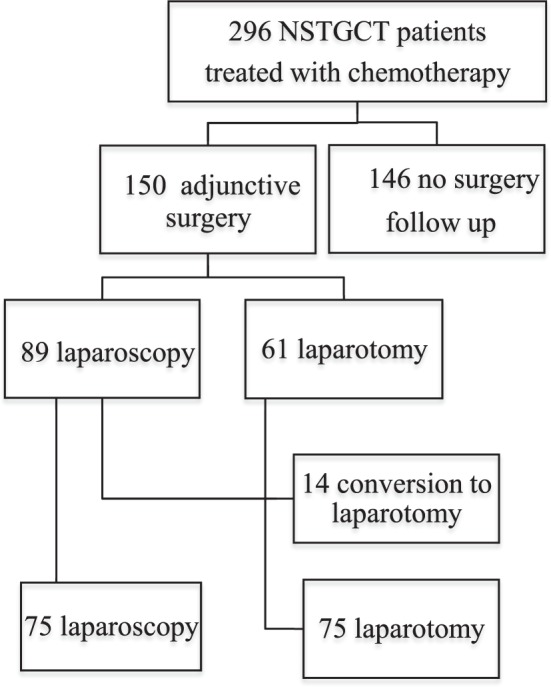
Patient flow chart of all 296 consecutive NSTGCT patients with disseminated disease treated with 3 of 4 courses of cisplatin based combination chemotherapy at the UMCG between 2005 and 2015.

A total of 296 disseminated patients with NSTGCT were treated at the Department of Urology, Surgical Oncology and Medical Oncology, of the UMCG between 2005 and 2015. Of these 296 patients, 150 underwent resection of RRTM after 3 or 4 cycles of cisplatin based combination chemotherapy. All patients were prospectively studied. Until 2004, the UMCG gold standard was RRRTM using a conventional midline laparotomy. The results of this policy with respect to the oncological, sexual, and psychosocial outcomes were previously described^4,5,12^. Laparoscopic resection of RRTM was introduced at the UMCG in 2004. The selection criteria for L-RRRTM were based on tumor size and the localization of the residual mass and were described previously in the pilot report^17^. In short, patients were candidates for a laparoscopic approach if the RRTM was less than 5 cm in diameter and located ventrally or laterally from the aorta, inferior caval vein, or iliac vessels. Incidentally, a slightly larger RRTM up to max 7 cm at a favorable para-aortic anatomical location was accepted for laparoscopic resection. In these cases, a higher risk for conversion was considered and discussed with the patient before the procedure. Patients with a RRTM posterior to the great vessels and/or a tumor mass larger than 5 cm were not considered candidates for laparoscopy. A flow diagram of the current series of 296 patients with NSTGCT scheduled for L-RRRTM or C-RRRTM is presented in Fig. 1. Also presented in Fig. 1 are the number of converted procedures from laparoscopy to an open procedure.

### Surgical procedure

The surgical procedure, excising only visible abnormal retroperitoneal tumor masses, has been extensively described previously in the feasibility report^17^. The laparoscopic resections were performed by 5 experienced laparoscopic surgical oncologists together with dedicated surgical oncology fellows. Key points regarding the laparoscopic procedure are that patients were positioned in the “French” or in a half-right lateral position depending on the localization of the RRTM^4,5,17^. Surgical resection comprised only the resection of the RRTM and conversion was performed in case of technical difficulties due to patient and/or tumor characteristics and/or complications.

Factors leading to conversion were classified into two categories; *reactive conversion* (RC), which is defined as one that follows an intraoperative event such as bleeding, and *pre-emptive conversion* (PC), which is defined as a conversion undertaken to avoid complications such as unclear anatomy, obesity, and a time-consuming laparoscopy procedure without any ‘surgical progress’ during the resection of the RRTM.

Conventional resection was performed with the same oncological principles, excising only visible abnormal retroperitoneal tumor masses^3–7^.

### Postoperative procedure

Direct postoperative follow up was performed at the UMCG by the Department of Surgical Oncology and long-term follow up by the Department of Medical Oncology according to the guidelines of the European Society for Medical Oncology (ESMO). Within this protocol, a monthly clinical and tumor marker evaluation was performed over the first year, followed by a gradually tapering schedule, with annual evaluations from years 5 to 10. Computed tomography was done 6, 12, and 24 months after complete resections and 6, 12, 24, and 60 month after incomplete resections.

### Statistical analysis and assessment of complications

A prospective dataset, including the previously described patients^17^, was constructed of all patients undergoing L-RRRTM or C-RRRTM from 2005 to 2015 comprising all patient and treatment-related information. Intra- and post-operative complications were categorized using administrative and electronic medical records. Patients were asked with regular intervals about symptoms of retrograde ejaculation during outpatient clinic visits and these events were recorded. Statistical differences between the two groups were analyzed and univariate analysis performed using the Fishers exact test and Mann-Whitney’s *U* test. Survival analysis was performed using the Kaplan-Meier method with the log rank test. All tests were double sided, and p values < 0.05 were considered to indicate significance.

## Results

### Pre-operative characteristics

The post-chemotherapy, pre-operative patient and tumor characteristics of the 150 patients (median age 27 [range 16–66] years) are presented in Table 1. The most common primary histology was embryonal carcinoma (n = 99, 66%). There were no statistically significant differences between the primary histology in the two groups except for embryonal carcinoma in the L-RRRTM group (p < 0.001). Patients with good prognoses according to the International Germ Cell Cancer Collaborative Group (IGCCCG), were more likely to undergo L-RRRTM than C-RRRTM (81% vs. 29%; p < 0.001). The majority of patients treated with L-RRRTM had clinical stage II disease (68 patients [77%] vs. 31 patients [51%] in the C-RRRTM group; p < 0.05).

**Table 1:** Preoperative patients’ and tumor characteristics.

Variable	L-RRRTM	C-RRRTM	p value
	N = 89	N = 61	
Age (median, range) yrs	27 (16–66)	28 (16–64)	0.11
**Histology compounds testis tumor; n (%)**			
Seminoma	28 (32)	13 (21)	0.29
Immature teratoma	31 (35)	11 (18)	0.07
Mature teratoma	46 (52)	28 (46)	0.69
Embryonal cell carcinoma	73 (82)	26 (43)	<0.001
Chorioncarcinoma	6 (7)	8 (13)	0.11
Yolk sac	41 (46)	17 (28)	0.05
**IGCCCG risk score before chemotherapy; n (%)**			
Good	72 (81)	18 (29)	<0.001
Intermediate	12 (13)	26 (43)	
Poor	5 (6)	17 (28)	
**Stage (Royal Marsden); n%**			
IIA	15 (17)	1 (2)	<0.05
IIB	41 (46)	11 (18)	
IIC	12 (14)	19 (31)	
III	2 (2)	13 (21)	
IV	19 (21)	17 (28)	
**Residual tumor location; n (%)**			
Para-aortic	61 (69)	25 (41)	0.001
Paracaval	9 (10)	11 (18)	
Inter-aorto/caval	16 (18)	21 (34)	
Iliacal	3 (3)	4 (7)	
Diameter RRTM mm (median range)	20 (5–70)	42 (11–220)	<0.001

L-RRRTM, laparoscopic resection residual retroperitoneal mass.

C-RRRTM, conventional resection residual retroperitoneal mass.

N/F, necrosis and/or fibrosis.

The most common residual tumor location was para-aortic (86, 57%); 69% in the L-RRRTM group versus 41% in the C-RRRTM group (p = 0.001). The median residual tumor size was significantly smaller in the L-RRRTM group; 20 (5–70) mm vs. 42 (11–220) mm in the C-RRRTM group (p < 0.001).

### Operative and outcome characteristics

Eighty-nine patients with a median RRTM mass of 20 (range 5–70) mm and for whom the vast majority (n = 72, 81%) belonged to the IGCCCG good risk category, were scheduled for laparoscopic resection of the RRTM with the intention of resecting it. Para-aortic RRTM location was found significantly more often in the group scheduled for L-RRRTM (61 vs. 25 in the C-RRRTM group; p < 0.05).

**Figure 2:**
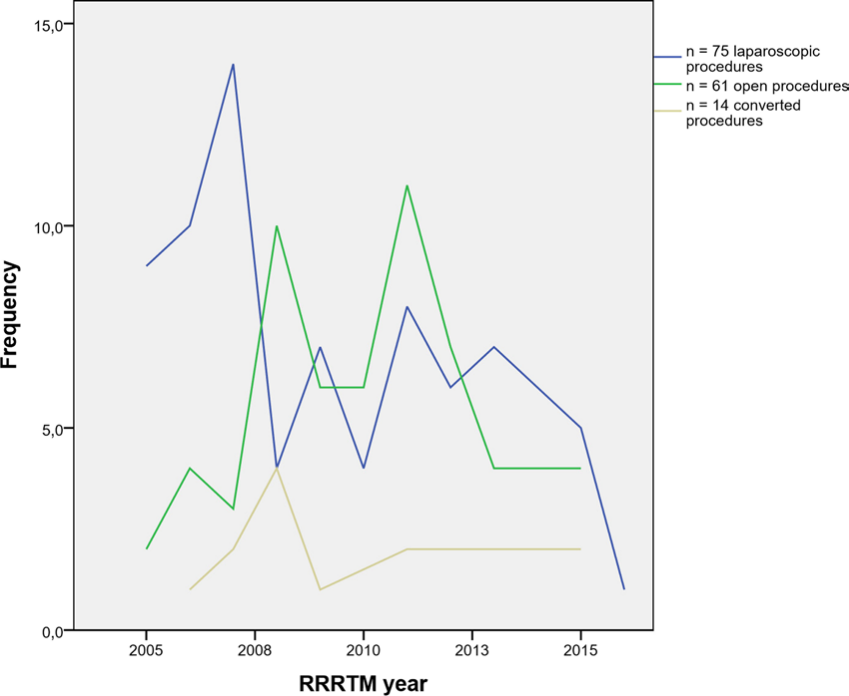
Graph showing number of procedures performed between 2005 and 2015.

In 14 of 89 patients (15%), a conversion to open, conventional surgery was necessary. As displayed in Fig. 2, the conversion rate did not change over the years. In 11 patients, pre-emptive conversions (12%) were performed due to technical difficulties (n = 7) and patient-related factors (n = 4). In 3 patients, reactive conversions (3%) were performed due to complications that occurred intraoperatively: ureter injury, aortic bleeding, and a bleeding of the inferior caval vein, respectively.

In more detailed analyses, operative outcome characteristics for the L-RRRTM (n = 89) and C-RRRTM (n = 91)are presented in Table 2. The surgical procedure was significantly shorter in duration in the laparoscopy group versus the open group (p < 0.001) with a median of 156 minutes versus 221 minutes.

**Table 2:** Operative Characteristics: 2005–2015 laparoscopic and conventional surgery.

Variable	L-RRRTM N = 89	C-RRRTM N = 61
Operative time; minutes, median (range)	156 (45–341)*	221 (95–792)*
Postoperative complications; n (%)	17 (19)	18 (30)
None	72 (91)	43 (70)
Wound infection	4	6
Chylous leakage	3	5
Pulmonary infection	1	0
Urinary complications	4	1
Haemorrhage/haematoma	1	0
Thromboembolism	1	0
Ileus	1	1
Retrograde ejaculation	2	5
Incisional hernia	0	0
**Residual tumor histology; n (%)**		
N/F	33 (37)	26 (43)
Teratoma +/−N/F	42 (47)	26 (43)
vital carcinoma +/−teratoma	14 (16)	9 (14)
Hospital stay; days, median (range)	2(1–13)*	6 (3–26)*

L-RRRTM, laparoscopic resection residual retroperitoneal tumor mass.

C-RRRTM, conventional resection residual retroperitoneal tumor mass.

*p < 0.001.

In the converted group of 14 patients median residual tumor size was 29 (16–70) mm, which was not significantly different from the median RRTM size in the whole laparoscopy group. Analysis showed, there were no significant differences in perioperative complications between the 89 patients scheduled for L-RRRTM (5 patients [6%]; 1 ureter injury, 4 patients with >500 mL blood loss) and the 61 patients scheduled for C-RRRTM (7 patients [12%]; 1 ureter injury, 6 patients with >500 mL blood loss) (p < 0.19). Seventeen postoperative complications (19%) occurred in those scheduled for L-RRRTM (n = 89), and 18 postoperative complications (30%) occurred in those scheduled for C-RRRTM (n = 61) (p = 0.14). Subgroup analysis showed that patients in the laparoscopically converted RRRTM (LC-RRRTM) and C-RRRTM groups were more likely to develop wound infections, chylous leakage, pulmonary infections, and retrograde ejaculation. There were 9 postoperative complications in the final L-RRRTM group (n = 75, 12%) versus 8 in the LC-RRRTM group (n = 14, 57%) (p < 0.001) and 18 in the C-RRRTM group (n = 61, 30%) (p < 0.05). Also, there were no significant changes in perioperative and postoperatieve complication rate in the period 2005–2015 in the L-RRRTM and C-RRRTM group.

The different histologies of the resected RRTM for both groups are presented in Table 2. The median operative time in the L-RRRTM group was 156 (35–341) minutes versus 221 (95–792) minutes in the C-RRRTM group (p < 0.001). The median length of hospital stay for the L-RRRTM group was 2 (1–13) day vs. 6 (3–26) days in the C-RRRTM group (p < 0.001). Excluding the 14 converted procedures, median hospital stay in the L-RRRTM group was 1 (1–5) day.

The median follow up for all 150 NSTGCT patients after RRRTM was 79 (range 2–144) months. Twenty-seven of 150 patients had recurrences (18%). Eight patients (9%) in the L-RRRTM group and 19 patients (31%) in the C-RRRTM group (p < 0.001) had recurrences. The oncological outcome is presented in detail in Table 3.

**Table 3:** Outcome characteristics: 2005–2015 laparoscopic and conventional surgery.

Variable	L-RRRTM N = 89	C-RRRTM N = 61
Follow up; months, median (range)	91 (7–144)^#^	70 (2–140)^#^
Recurrence; n (%)	8 (9)^a^	19 (31)^a^
Survival status; n (%)		NS
No evidence of disease	87 (98)	54 (88)
Alive with disease	1 (1)	1 (2)
Died of disease	1 (1)	5 (8)
Died of other causes	0	1 (2)

L-RRRTM, laparoscopic resection residual retroperitoneal tumor mass.

C-RRRTM, conventional resection residual retroperitoneal tumor mass.

NS: non significant, ^#^p < 0.05, ^a^p < 0.001.

The histopathology of the 25 resections for recurrent disease showed necrosis/fibrosis in 6 (22%) patients, viable germ cell cancer in 7 patients (26%), teratoma in 12 patients (44%). In 2 patients resection was not performed and therefore histology not available. Six patients with viable germ cell cancer at the time of recurrence were in the C-RRRTM group versus one patient in the L-RRRTM group. Three of these 6 patients had also viable germ cell cancer in their initial RRRTM pathology results. A detailed overview of the 27 patients with recurrences is summarized in Table 4.

**Table 4:** Characteristics of 27 nonseminomatous testicular cancer patients with relapse.

Pat (No)	Age (yrs)	Side primary tumor	Histology*	Stage Royal Marsden	IGCCC	Diameter RRTM (mm)	Location RRTM	Histology ϕ	FU since RRRTM(mo)	Months until first relapse	Treatment relapse	Histology relapse surgery ϕ	Outcome
**L-RRRTM**													
1	33#	Right	3,4	IV	1	11	interaortocaval	N/F	113	9	CTX, RRRTM	N/F	NED
2	30	Right	1,2	IV	1	17	interaortocaval	T,VT	98	11	RRRTM	T	NED
3	27	Left	1,3	II	1	46	paraaortal	T,N/F	97	61	CTX	*N/A*	DOD
4	29	Right	1,2	II	1	21	interaortocaval	VT	18	14	CTX, RRRTM	VT	AWD
5 ͨ	41#	Right	1,2,3,4	II	1	21	paraaortal	T,VT	113	46	RRRTM	T	NED
6 ͨ	37	Left	1,4	IV	2	29	paraaortal	N/F	106	1	CTX, thoracotomy	N/F	NED
7 ͨ	20	Left	1,2	II	1	16	paracaval	N/F	96	9	RRRTM	N/F	NED
8 ͨ	27	Left	1,3	II	1	29	paraaortal	N/F	51	36	RRRTM	T	NED
**C-RRRTM**													
9	27	Left	1	III	1	17	paraaortal	T	109	27	RRRTM	T	NED
10	37	Right	6	IV	3	15	paracaval	T	107	96	RRRTM	VT	AWD
11	19	Left	1,2,3	IV	2	160	paraaortal	T	116	2	Thoracotomy	T	NED
12	38	Right	3	III	2	15	paraaortal	N/F	34	6	Thoracotomy	N/F	NED
13	28	Right	3	IV	2	80	interaortocaval	T,N/F	105	5	CTX, resection mass neck	T	NED
14	26	Left	1,3	II	2	30	interaortocaval	T	96	13	RRRTM	T	NED
15	24	Left	1,2,3	III	3	31	paracaval	T/VT	26	9	Thoracotomy	VTª	DOD
16	53	Left	5	II	2	80	iliac	VT	7	1	No treatment options	*N/A*	DOD
17	33	Left	2,3	III	2	84	interaortocaval	T/VT	90	5	Thoracotomy	T	NED
18	22	Left	5	III	2	65	paraaortal	T/VT	89	52	CTX, RRRTM	VT,T	NED
19	21	Right	1,2,3	II	1	47	iliac	T	68	10	CTX, RRRTM	T	DOO
20	31	Left	1	III	1	69	interaortocaval	T	89	3	RRRTM	T	NED
21	28	Right	1,2	IV	3	16	paraaortal	N/F	67	7	CTX, RRRTM	N/F	NED
22	28	Left	3	II	1	35	paraaortal	T	19	7	CTX, RTX RRRTM	VTª	DOD
23	23	Left	3	II	3	220	paraaortal	T,VT	58	8	CTX, RRRTM	VT	NED
24	24	Right	3	III	3	220	interaortocaval	T,N/F	45	4	Thoracotomy	T	NED
25	20	Right	1,2	II	1	29	iliac	T	33	10	RRRTM	T	NED
26	37	Left	3,4	III	1	96	paraaortal	T,VT	25	8	Thoracotomy	N/F	NED
27	23	Left	1,2,3	IV	2	25	paraaortal	T,N/F	103	103	RRRTM	VT, T	NED

L-RRRTM: laparoscopic resection residual retroperitoneal tumor mass, C-RRRTM: conventional resection residual retroperitoneal tumor mass, ͨ :converted procedures.

^#^Previously reported^17^ *Histology: 1: embryonal carcinoma, 2: yolk sac tumor, 3: teratoma, 4: seminoma, 5: burnout lesion, 6: unknown.

^ϕ^Histology: N/F: necrosis/fibrosis, T: teratoma, VT: viable germ cell cancer, ªEmbryonal rhabdomyosarcoma (transformation from mature teratoma).

CTX: chemotherapy, RTX: radiotherapy. NED: no evidence of disease, AWD: alive with disease; DOD died of disease; DOO died of other causes.

Of the 27 patients with recurrent disease, 26 (96%) received (combined) treatment; 16 (59%) had surgery alone, 9 had (33%) systemic chemotherapy plus surgery, 1 (4%) had only systemic chemotherapy, and 1 (4%) had no treatment.

**Figure 3:**
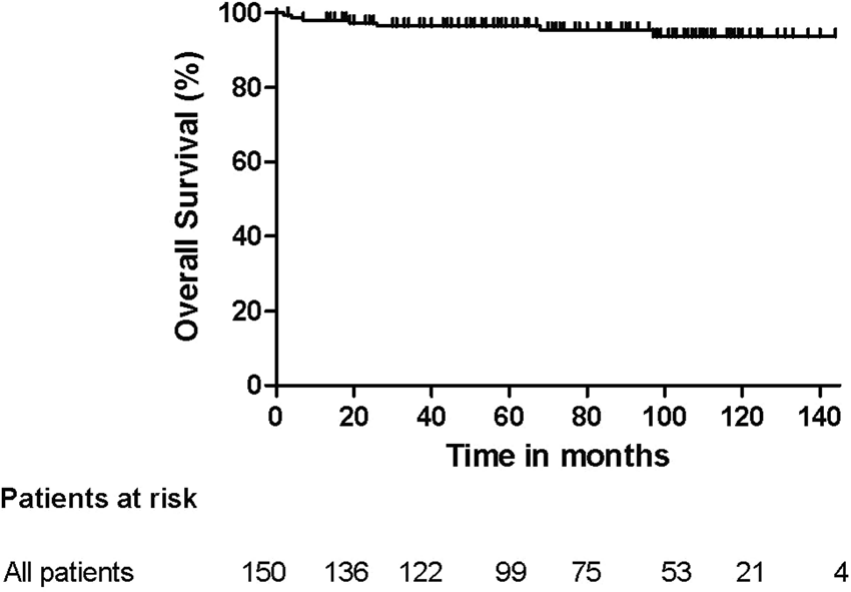
Overall Survival after RRRTM: all patients.

**Figure 4:**
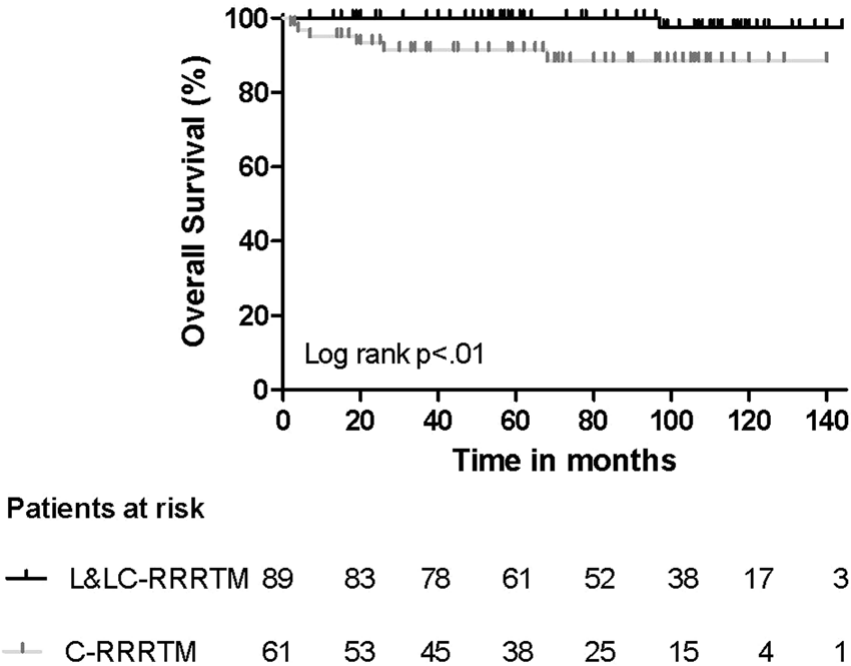
Overall Survival after L- and LC-RRRTM versus C-RRRTM: Log rank p < 0.01.

**Figure 5:**
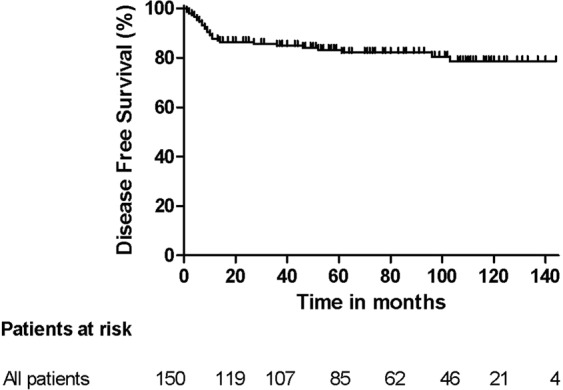
Disease Free Survival after RRRTM; all patients.

**Figure 6:**
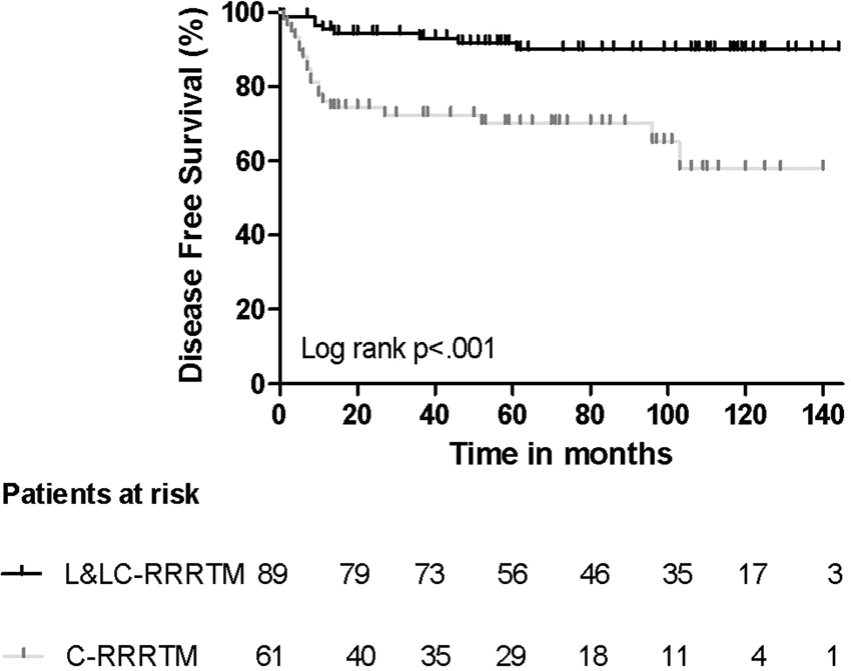
Disease Free Survival after L- and LC-RRRTM versus C-RRRTM: Log rank p < 0.001.

The disease-free survival (DFS) and overall survival (OS) of all patients and those scheduled for L-RRRTM (n = 89) and C-RRRTM (n = 61) are presented in Figs 3–6. Significant differences were found in OS and DFS between the initially scheduled and final treatment groups.

## Discussion

This is currently the largest series published in literature with respect to laparoscopic resection of RRTM after cisplatin based combination chemotherapy for metastatic NSTGCTs. In cases of RRTM after chemotherapy for metastatic NSTGCT, adjunctive resection of all residual tumor mass is an essential part of the combined treatment to cure these patients^3–7^. In an earlier pilot study, the Groningen study group showed that the laparoscopic approach was feasible in properly selected patients with advanced NSTGCT, with surgical resection comprising of only the resection of the RRTM^17^. However, the study included a relatively small cohort of patients, and longer follow up data are lacking with respect to disease free survival. The current report includes the previously described patients^17^.

Since the introduction of laparoscopic RPLND in 1992, and with the further development and increasingly routine use of laparoscopic techniques, the minimally invasive approach is gaining interest in the treatment of mainly lower-stage testicular cancer. The nine studies published to date are summarized in Table 5 ^14,18–25^.

**Table 5:** Comparison series of laparoscopic RRRTM.

Series	Year	N	Stage	RRTM size (cm)	Conversion rate N (%)	PC N (%)	RC N (%)	Peri-operative Complications N (%)	Post-operative Complications N (%)	Mean OR time (min)	Mean hospital stay (days)	Follow up mean (range) (months)	Recurrence N (%)
Rassweiler^19^	1996	8	2 II B 7 II C	—	6 (75)	5 (83%)	1 (17%)	0 (0)	1 (13)	357	7	27 (4–43)	none
Steiner^20^	2004	68	10 IIA 43 IIB 15 IIC	—	0			0 (0)	28 (17)	243	4	58 (3–121)	1 (1)
Albqami^14^	2005	59	43 IIB 16 IIC	—	0			9 (15)	11 (19)	234	4	53	1 (2)
Calestroupat^18^	2009	26	16 IIA/B 13 II C	3.4 (2–6)	5 (2.6)	1(20%)	4 (80%)	9 (35)	none	183	5	27 (14–36)	none
Busch^21^	2012	43	26 II 20 III	2.2	3 (6.5)	0	3 (100%)	21.7	—	212	6	30 (12–47)	4 (8.6)
Aufderkamm^24^	2014	19	5 IIA 7 IIB 5 IIC 2 III	3.87 (1.5–9.7)	0			0	2 (10.5)	212	6	18 (12–90)	0
Gaya^23^	2015	15	2 I 9 II 4 III	4.7	2 (13)	0	2 (100%)	5 (33)	—	294	5	29 (1–79)	none
Nicolai^22^	2016	67	14 IIA 41 IIB 7 IIC 5 III	27 (15–31)	3 (4.5)	2 (67%)	1 (33%)	1 (1.5)	3 (4.5)	234	3	21	none
Nakamura^25^	2016	14	14 IIA/B	25 (18–30)	0			0	7 (50)	439	1	36	none
UMCG series	2017	89	15 IIA 41 IIB 12 IIC 2 III 19 IV	19 (5–57)	14 (16%)	11 (78%)	3 (22%)	5 (6%)	9 (12%)	148	1	79 (7–144)	3 (4%)

In the present study, analysis of the L-RRRTM group was done in parallel with that of the C-RRRTM group. The L-RRRTM group consisted of successfully performed laparoscopies (n = 75) as well as the 14 converted procedures (LC-RRRTM) and the C-RRRTM group (n = 61) included the patients who underwent an open procedure to resect the RRTM. Statistical analysis performed between these groups should be evaluated in a more descriptive fashion since this was not a randomized trial and selection bias indisputably exists, leading to confounding factors such as the IGCCCG category and amount of residual disease after chemotherapy.

A significant difference in median RRTM diameter was found between the group scheduled for L-RRRTM (20 [range 5–70] mm) versus the group scheduled for C-RRRTM (42 [11–220] mm; p < 0.001). This significant difference is in line with the study by Busch *et al*. where the RRTM diameter was 22 mm in the L-RRRTM group versus 68 mm in the C-RRRTM group^21^. Para-aortic RRTM location was found significantly more often in the L-RRRTM group than in the C-RRRTM group. Resection of a residual tumor located para-aortically is technically less challenging than resection of a RRTM at an inter-aortocaval or para-caval location.

Although the conversion rate in the literature varied in the past from 0% to up to 75%, the current UMCG conversion rate of 15% is in line with current conversion rates varying from 0% to 13%^14,18–25^. In general, the conversion rate decreases with surgical experience but increases with the size of the RRTM. The UMCG conversion rates in the current series are slightly higher than that of the previous pilot report (15% vs.10%) mainly due to pre-emptive factors such as technical difficulties and patient related factors (50% vs. 29%) and in lesser extent due to reactive factors (21%). Most indications for conversion described in literature are reactive in nature in contrast to this study. For example, obese patients were not always excluded for laparoscopy. A reactive conversion was required in only 3 patients.

Classical RPLND or RRRTM is associated with substantial morbidity. No significant difference was found in the perioperative complication rate between L-RRRTM and C-RRRTM group (6% and 12%). In contrast, in an open and laparoscopic cohort, Nicolai *et al*. documented higher intraoperative laparoscopic complication rates of 37.9% vs. 21.8% (NS)^22^. A smaller laparoscopic cohort showed a laparoscopic intraoperative morbidity of 33%^23^. The UMCG series showed that concerning the postoperative morbidity there were no significant differences in patients undergoing a L-RRRTM (19%) compared to those receiving the C-RRRTM(30%). Although there is a tendency for a higher postoperative complication rate in patients undergoing an open procedure which can be explained by the more extensive retroperitoneal disease in these patients and/or the anatomical location of the residual tumor mass. In the same manner, Nicolai *et al*. also documented no significant differences between these groups; 14% vs. 9%^22^.

The median operative time in the present study was significantly shorter in the L-RRRTM group versus the C-RRRTM group. This is to be expected since the latter group had more extensive disease and larger RRTM volume; the median residual tumor mass was 20 (range 16–66) mm in the L-RRRTM group, and 42 (range 11–220) mm in the C-RRRTM group. In contrast, other more recent studies reported longer operative times for laparoscopic procedures of 234, 294, and 439 minutes^23–25^. Nicolai *et al*. reported equivalent operative times (212 vs. 232 min, p = 0.3)^22^. A study originating from Japan reported not only longer operative times in the laparoscopic group, but also in the open group (439 vs. 408 minutes)^25^.

As expected, the L-RRRTM group required a significantly shorter hospital stay compared to the C-RRRTM group (medians of 2 day vs. 6 days; p < 0.001). In contrast to the short hospital stay for the laparoscopy patients in this series, the median hospital stay in other studies was longer after L-RRRTM, ranging from 3–7 days (Table 5). Nicolai *et al*. also compared the L-RRRTM group versus the C-RRRTM group and documented a median hospital stay of 6 vs. 11.5 days (p < 0.01)^22^.

Thus far, there are no long-term outcome data in advanced nonseminomatous germ cell tumors comparing laparoscopic RRRTM to conventional RRRTM. After a median follow-up of 79 months, 27 patients (18%) developed recurrent disease; 19 in the C-RRRTM group (31%) and 8 in the L-RRRTM (9%): 4 of these 8 recurrences occurred in patients who underwent converted procedures. Reasons for conversion in these 4 patients were aortic injury, injury to the caval vein, and two technical difficulties due to extensive fibrosis which could be indicative of more extensive disease. Other laparoscopic surgeries for resection of residual masses also showed low recurrence rates (Table 5). However, most reports consist of only small patient series and predominantly include patients with good prognosis disease and small residual tumor masses, with a limited follow-up duration^14,18–20^.

When selecting patients for L-RRRTM, factors other than merely the size of the mass are important. Selection criteria are not absolute and these criteria are dynamic; first, in relation to developing skills and second, the anatomical site of the residual mass. Large residual masses greater than 5 cm might be eligible for a laparoscopic approach based on the laparoscopic experience of the surgical oncologist and the anatomical location.

Surgeons can now also perform robotic-assisted RRRTM^26,27^. Within a few years, robotic surgery might extend the indication for L-RRRTM, even for more challenging RRTM. L-RRRTM meets or exceeds the results from most open conventional surgeries and should always be considered as a viable alternative in the resection of residual tumor mass after cisplatin-containing chemotherapy for locally advanced testicular cancer, offering less morbidity, a favorable cosmetic outcome for the patient, and a shorter hospital stay. The study showed that laparoscopic resection of RRTM in proper selected and well-defined patients is a feasible, non inferior procedure to a conventional laparotomy for the resection of low volume RRTM with a longterm recurrence rate of 9%. Although a randomized trial comparing both treatment modalities, L-RRRTM vs. C-RRRTM, is the gold standard to proof the superiority of this minimally invasive approach for low-volume disease and therefore the results should be interpreted with caution. A multidisciplinary expert testicular cancer team with an experienced (oncological) laparoscopist is essential to achieve these peri-operative and longterm results and this has to be taken into account in the discussion of treatment options, L-RRRTM vs. C-RRRTM, with the testicular cancer patient.

In conclusion, with a robust sample size and a median follow-up duration of more than 6 years, this study confirms that laparoscopic resection of well-defined RRTM after cisplatin combination chemotherapy for metastatic nonseminomatous testicular cancer is a feasible procedure and appears to be an oncologically safe option in appropriately selected patients, offering oncological non inferior results compared to conventional surgery, with less morbidity, a shorter hospital stay, and a favorable cosmetic outcome for the patient with testicular cancer. Despite the fact that technical boundaries are gradually expanding due to developing skills and technical innovations it can be stated that residual masses up to 5 cm and located para-aortically seem suitable for laparoscopic resection. These surgical procedures, L-RRRTM and C-RRRTM, should preferably take place in a high volume tertiary referral centers for the treatment of testicular cancer.
